# Adolescents With Cancer Need Trustworthy Information and Prefer to Receive It From a Human Source Rather Than From the Internet: A Qualitative Study

**DOI:** 10.3389/fpsyg.2021.746810

**Published:** 2021-11-22

**Authors:** Irit Schwartz-Attias, Haya Raz, Tamar Natanzon-Bracha, Adi Finkelstein, Shulamith Kreitler

**Affiliations:** ^1^Meir Medical Center, Kfar Saba, Israel; ^2^Jerusalem College of Technology, Jerusalem, Israel; ^3^Schneider Children’s Medical Center, Petach Tikva, Israel; ^4^The School of Physiological Sciences, Tel Aviv University, Tel Aviv, Israel

**Keywords:** information needs, adolescents with cancer, sexuality, telling the truth of cancer, information resource

## Abstract

**Background:** In pediatric cancer, the legal obligation to provide information is usually toward the parents who are the authorized signatories of the informed consent form. It is now known that aside from providing information to the parents, it is also very important to provide information to the children and adolescents themselves. The question is how the adolescents relate to this. What information do they already possess and what would they like to know? Would they wish to hear the truth in all situations and at what stage? What are their preferred sources of information?

**Method:** A qualitative study that included in-depth interviews with 19 adolescents with cancer, aged 8.5–18, who were receiving active treatments and had been diagnosed at least 1 month previously. The interviews were guided by 15 open-ended questions.

**Findings:** The analysis of the interviews indicated that adolescents know quite a lot about the course of their disease and the information they lack is mainly etiological. The participants reported a lack of knowledge concerning sexuality and a sense of discomfort talking about it, leaving them with open questions. They all claimed that it is important to tell the truth: “Even if the truth is difficult, it is important to tell it.” The participants reported that information can be scary, so it must be structured and adapted to the age and emotional readiness of the individual. Most of the participants prefer not to use the internet as an information resource due to the profusion of stressful and non-adapted information.

**Conclusion:** Adolescents with cancer need trustworthy information and prefer to receive it from a human source rather than from the internet. Not telling the truth can lead them to feel fear and loneliness. The medical staff must operate in sensitive and creative ways to provide adolescents with access to information on various subjects, including sexuality, which they are ashamed to talk about, leaving them with a sense of shame and a lack of knowledge in this area.

## Introduction

In the patient-centered approach, communication skills constitute significant milestones (Zanon et al., 2020). This approach advocates sharing with the patient all details of the diagnosis, the treatments, and their consequences (Ali, 2017). There is a great deal of information on the significance of adapting the manner of communication of the medical staff to the values and preferences of the patient (Kissane et al., 2010; Brown et al., 2016). Many studies conducted in this area found a correlation between effective personally adapted communication and compliance with treatment and better treatment outcomes (Ali, 2017). Legally, when diagnosing pediatric cancer, the responsibility to provide information regarding the diagnosis and treatments falls on the guardian. The most common guardians are the parents, who are the authorized signatories of the informed consent form for patients under 18. Aside from providing information to the parents, it is very important to provide information regarding the diagnosis and planned treatment to the children and adolescents themselves (Bahrami et al., 2017; Stein et al., 2019). The ability of children to understand the diagnosis and the treatment protocol depends on many factors, including their developmental stage. A study conducted among survivors of childhood cancer found that adolescents who had received structured information at the diagnosis stage (adapted information provided by a member of the medical staff not long after the diagnosis) displayed fewer psychological impairments further on in life than those who had not received such information (Raz et al., 2016).

### Information for Adolescents – What Do They Want to Know

Communication with adolescents with cancer is extremely important and can affect their adjustment to the diagnosis and treatments, their satisfaction with the treatment, their decision-making process, and further, their life as healthy individuals in the community (Raz et al., 2016; Bahrami et al., 2017; Bibby et al., 2017). Using various research methods, Decker et al. (2004) investigated the information that adolescents wish to receive about their cancer and how they are affected by the information or the lack thereof. In the quantitative part of the study, considerable significance was attributed to the need for information on handling medical procedures, relations with friends and family, returning to school, and completion of treatment. In the qualitative part, additional issues that occupy affected adolescents were uncovered, including types of treatment and side effects, uncertainty regarding the future, as well as social and emotional matters. In light of the need for information on different topics, including psychosocial aspects, the researchers concluded that ongoing informational support should be provided to the affected adolescents and that they should be provided with information regarding their illness, its treatment, and the physical and emotional implications of this type of challenge, from the moment of diagnosis (Decker et al., 2004).

In the two decades that have elapsed since then, the knowledge base regarding the unique needs of adolescents with cancer has not grown considerably. In their extensive literature review, Bibby et al. (2017) found that most publications in this area do not relate specifically to adolescents with cancer. The review, which included research on adolescents and young adults (AYA) aged 15–30, included 45 articles, where only about one-quarter of them referred to adolescents. The same review indicated that adolescents with cancer report a need for age-adjusted information concerning diagnosis, treatments, maintaining fertility, a healthy lifestyle, and recovery. In addition, adolescents reported that receiving information helps them prepare for the future better, while the lack of information is associated with a sense of stress and dissatisfaction with treatment (Bibby et al., 2017). A qualitative study conducted in Iran among 12 adolescents, aged 15–20, with cancer found that all the participants reported that the information they had received directly about their illness was deficient and that most of the information had been provided to their parents. The adolescents had (many) unanswered questions and were unable to find a reliable source for obtaining answers (Bahrami et al., 2017).

### Sexuality Among Adolescents

Sexuality constitutes an important aspect in the development of adolescents and in their process of entering the adult world, and it is associated with their well-being and quality of life (Laumann et al., 1999). The literature indicates that the topic of sexuality continues to occupy the thoughts of many adolescents despite their condition (Veneroni et al., 2020).

Nevertheless, the topic is often considered taboo by health care providers, who are uncomfortable to raise and discuss it openly with adolescents (Graugaard et al., 2018; Veneroni et al., 2020). In their reports, adolescents with cancer raise difficulties related to sexuality, such as altered body image, diminished self-value, decreased sexual drive, the concern of the spouse regarding having sex as before the diagnosis, as well as other concerns (Graugaard et al., 2018; Veneroni et al., 2020). It is concerning that adolescents report a lack of opportunity to discuss these topics. A study conducted among 66 adolescents with cancer, aged 16–24, in Italy, found that 39% of the participants reported that health care providers had never spoken to them about these topics, while 17% claimed that these topics had been discussed with them to a limited extent. Eighty percent of the participants thought that these topics should be given more room in the therapeutic discourse (Veneroni et al., 2020).

### Telling the Truth From the Beginning

The issue of telling the truth to patients is the topic of many ethical discussions. Davies (2020) deals with whether it is always right, to tell the truth, what to say and how, and what happens if patients 18 years and older do not want to know, or if the information might harm them. Additionally, what happens when the patient is a minor (under 18) and the obligation of providing the information to the patient is entrusted to the guardian (Zanon et al., 2020). According to the directives developed by the International Society of Paediatric Oncology (SIOP), children and adolescents under 18 with end-stage cancer should be given as much information as possible about their condition in accordance with their age and developmental stage, “Depending on age and level of development, the child should also be involved in the decision, with older children especially participating more actively” (Masera et al., 1999, p. 46). The professional directives of the SIOP from 2009 also state that sick children and adolescents under 18 have a basic right to receive adapted information (Kowalczyk et al., 2009). But these directives are nonetheless very general and leave much room for personal and professional interpretation. A study conducted among 203 AYA aged 15–29 found that patients who received an extensive explanation regarding their prognosis reported more trusting relations with the health care providers, mental calmness, and more hope (Mack et al., 2018). A qualitative study held in Sweden among adolescents with cancer, aged seven to 17, found that they want to hear the truth about their condition, including receiving difficult information. All the participants claimed that no matter how tough the information, they want to know it, but it is important for them that during the conversation they are not denied hope (Jalmsell et al., 2016).

### Information Resources Among Adolescents With Cancer

Adolescents raise varied and diverse needs for information. The differences in the consumption of information may be related to age, the stage of the illness, type of disease, and resilience (Lea et al., 2018). One of the challenges confronting health care providers of adolescents with cancer is providing as much accurate and personally adapted information as possible. Each adolescent has different information needs in terms of the content and scope of information. For example, some adolescents will need information related to sexual relationships while others will need information related to obtaining a wig. Some adolescents need broad information and some need limited and superficial information. Naturally, some patients, including children and teens, will seek and gather information on their illness from their surroundings and according to their cognitive abilities. When diagnosing cancer in childhood or adolescence, parents themselves may be in a state of trauma and therefore the responsibility of delivering professional and reliable evidence-based information to sick children and adolescents falls to the medical staff (Zanon et al., 2020).

The internet is an important, readily available, and integral source of information in the life of adolescents, however, despite its advantages this information platform also has its shortcomings. A study conducted among 21 adolescents, aged 13–24, with cancer, found that searching for factual information about their cancer diagnosis, prognosis, treatment, tests, and procedures is complex and problematic. Some young people do not use this resource at all, and some express uncertainty and difficulties regarding the validity and reliability of the information and the professional jargon (Lea et al., 2018). Others report various emotional consequences such as fear, concern, and anxiety.

A study by Raz et al. (2016) examined the impact of the type of information on the emotional state of convalescents. Adolescents who reported that the information they received during their illness had been provided by the medical staff in an orderly manner demonstrated better scores for quality of life, psychological pain, and tolerance for psychological pain than convalescents who reported having obtained this information on their own.

The current study is a pioneer study conducted in Israel and its main purpose was to illuminate the existing and missing types of information and the preferred information resources among adolescents with cancer in active stages of treatment, using qualitative research methods. The study focused on adolescents with cancer, aged 8.5–18 years, an age group considered psychologically unique (Raz et al., 2016; Bahrami et al., 2017; Sawyer et al., 2018). Adolescence is characterized by the transition from childhood to adulthood when adolescents are engaged in seeking independence and establishing their personal and unique identity among other things while demonstrating introspective, abstract, and operational reasoning (Nixon, 2014). Therefore, the question is: What information would adolescents like to receive in the different stages of their illness, what are their preferred information sources, and how would they like to receive the information. In the current study, while receiving treatment, adolescents were asked what information they would like to receive from the moment of diagnosis, what are information resources they use, and what issues occupy them. According to the literature, this important topic has yet to be sufficiently investigated. Observing the life of affected adolescents and becoming familiar with their illness-related content worlds can help health care providers maintain evidence-based communication that may provide a better response to the unique information needs of the former regarding diagnosis and treatment and thus, improve their present and future psychosocial outcomes.

### The Research Questions

The main questions we sought to explore in this study were:

(1) What do children and adolescents know about their illness, by age?
(2) What would children and adolescents want to know in general or more than they know at present?
(3) What are the information resources they use and that they would recommend for others in their condition?

## Materials and Methods

### Participants

The study included 19 adolescent cancer patients (Table 1) treated at a tertiary pediatric medical center in Central Israel. The inclusion criteria were age ≤18 and ≥8 years, diagnosed with cancer, receiving chemotherapy, and speak Hebrew. Accordingly, the exclusion criteria were as follows: older than 18 or younger than 8, with no cancer. All the participants were Jewish, four reported that they keep a secular lifestyle, and the rest were traditional, religious, or ultra-Orthodox. The proportion of secular participants in the current study is low relative to their proportion in the Israeli population at large.

**TABLE 1:** Characteristics of the 19 participating adolescents.

		*N*
Gender	Male	10
	Female	9
Age	8.5–11.5	3
	12–15	5
	15.5–18	11
Diagnosis	ALL	8
	Lymphoma	3
	Ewing sarcoma	2
	Optic glioma	2
	Rhabdomyo sarcoma	1
	AML	1
	Endometrial carcinoma	1
	Aplastic anemia	1
On active treatment	Yes	19
	No	0

*ALL, acute lymphoblastic leukemia; AML, acute myeloid leukemia.*

The participants were recruited using a convenience sampling method. The consent process consisted of three parts - The first part included an explanation provided to the parents about the study. The second part included contacting the children whose parents had given their consent to participate in the study. The children received the same explanation as their parents did and upon expressing their consent, the interview phase began. In the interview phase, the researchers first contacted the 23 parents of the adolescents with cancer who had expressed consent for their child to participate in the study. Of those, only 19 children agreed to participate (82% response rate). The research participants were highly satisfied with the interview and said that they feel like ambassadors and are grateful for the opportunity and privilege of helping advance knowledge in the field for the benefit of future patients.

### Research Tool and Data Collection

A semi-structured interview following an interview guide was developed according to the main research questions. The interview guide consisted of 15 open-ended questions (Table 2) inspired by previous studies (Raz et al., 2016; Bahrami et al., 2017). At the beginning of the interview session, it was clarified to the participants that they could choose whether to answer the questions or not and that they could add information that matters to them even if not asked for. The face-to-face interviews took place in a private room, in the presence of the interviewing nurse and the adolescents, for about 45 min. To avoid a sense of threat or pressure on the children, the interviews were not recorded but the answers of the children were manually transcribed by the interviewers.

**TABLE 2:** Fifteen questions that guided the study.

Categories	Question No.	Content
Your illness	1	What do you know now about your illness?
	2	Would you like to know more about your illness than you know now?
	3	What kind of information would you like to be given about your illness?
	4	What do you think you should know about your illness?
Your treatments	5	What do you know now about your treatments?
	6	Would you like to know more about your treatments than you know now?
	7	What kind of information would you like to be given about your treatments?
Other illness	8	What do you think a child/adolescent should know about his illness?
	9	What kind of information is usually given to children and adolescents regarding their illness?
	10	What do you think that children and adolescents in the ward know about their illness?
Other illness and treatments	11	In your opinion, how does the information that children and adolescents receive about their illness and treatments, affect them?
Support resources	12	Who mostly helps you?
	13	What most helps you?
Sources of information	14	From whom did you get the information about your illness and treatments? (1) ___________; (2) __________
	15	Where do you search for information about your illness and treatments? (For example internet, parents, nurses, doctors, etc.)

### Ethical Considerations

The study was approved by the Helsinki Committee at the Rabin Medical Center and all the procedures required for this study were completed.

### Data Analysis

The data were analyzed using conventional qualitative content analysis (Hsieh and Shannon, 2005), where codes were defined during the data analysis and derived from the data. The analysis was done inductively, manually, imposing no categories or theoretical perspectives in advance. The analysis began by reading all the transcripts three times, to get a sense of all the data. Then the categorization guidelines of Hsieh and Shannon (2005) were followed. The recommendations of Morse (2015) for determining rigor in qualitative inquiry were maintained.

The first and second authors, who have rich experience in qualitative research, conducted the analysis. The analysis began by creating an initial coding scheme which was done separately by each member of the research team, which included nurses, a psychologist, and a sociologist. After reaching an agreement on the initial coding scheme, the first and second authors continued to the following stage of the analysis. The next step was to organize groups of codes into meaningful categories and subcategories, develop definitions for each category, and identify examples from the data. The team met several times to debrief and discuss the emergent categories and subcategories in-depth.

In the “Results” section, only a full description of the findings is given, as is customary in qualitative methods, and pseudonyms are used, besides the age of the child, for example (Amir, 17) or Amir (17).

## Results

### The Information That Children and Adolescents Have About the Disease, Treatments, and Side Effects

When asked about what they know about their illness, the participants could answer in their own words relating to their cancer diagnosis, for example, “a problem with cells” (Johnny, 12). Despite the variety in the level of specification/sophistication of the answers, they were able to correctly identify their organ or body system affected by the illness, for example, the blood or pelvis. “I have acute leukemia, a blood cancer. A condition where T cells in the bone marrow remain young and divide quickly. They occupy the bone marrow and it keeps other cells from thriving” (Jonathan, 16). Eight of the participants (*N* = 19) noted the name of the medical diagnosis or used the term “tumor” or “cancer.”

The participants knew what treatments they were receiving and what they would be receiving in the future: “I know I have 17 treatments; then surgery and then 11 more treatments” (Noa, 13). They recognized the names of the medications and the reason for taking them, as well as their side effects: “About the treatments, I know it’s chemo, that it destroys the healthy cells as well, and that it has side effects: hair loss, nausea, loss of taste” (Rachel, 15).

### Children and Adolescents Want to Know More About the Cause of Illness and the Treatments

Some of the participants said they need to understand the cause of their illness: “I would like to know why. How did it suddenly appear?” (Amir, 17); “When I arrived, they didn’t explain to me about the disease. I wanted to know what was causing it” (Dina, 17). Five participants were interested in what they could do to heal faster, for example, would a change to their nutrition or lifestyle help?

Nine participants said that it is important to share information with children because it helps them cope. They said that at first, they were apprehensive about the information, but ultimately it was reassuring: “At first, it was hard to accept it [the information], but it was good that I heard it.” (Ruth, 13). Dan (12) mentioned that “It is very important to know about the disease. It helps cope.” He shared that he wanted to know what caused the illness and how it would affect him at school. However, he would not like to know more about the treatments. Unlike him, most of the participants wanted to understand the treatment plan: “I need to know what I’m going through. not just come to the hospital and get the treatment” (David, 15).

The participants did want to know more, however, about the procedures and the side effects of the medications: “[I’d like to know more about] the side effects [of] the medications. to know more about the treatment itself rather than the disease” (Edna, 16). They said that they would rather receive the full information than discover it by themselves: “They didn’t **explain** to me when they inserted the port. I saw a video, but they didn’t really **explain**” (Ruth, 13). “I didn’t know about Ifosphamide [Chemotherapy drug]. I had side effects, but I didn’t know I would get so thin. I’d like to know that in advance… to know all the side effects” (Eli, 18).

Only two participants spoke openly about the lack of knowledge concerning sexuality and intimate relationships given their illness, and their need for information and support: “I would like to know what I am allowed, what I am not allowed to do (with my girlfriend) – I asked my mother and she didn’t know. I would like to know more in terms of my relationship. What’s allowed? It feels like I have no one to talk to about it. Although I would like to talk about it” (Eli, 18).

### Some Children and Adolescents Want to Get Any Kind of Information Even If It Is Intimidating, and Some Avoid It

The participants expressed no desire to know their prognosis or their chances of recovery, with one exception: “How did the illness develop? How many cases are there worldwide (because it is rare) and what is the chance of recurrence?” (Ron, 16).

The participants hardly talked about death and dying: “There are kids that may be scared by it (the information) and kids for whom it is a relief. For me, it’s a kind of relief. At first, I thought you could die of it, but my condition was not bad. You can take care of yourself and stay alive. [My] parents explained it and it was reassuring” (Hope, 12). Only one participant talked about a life-threatening illness: “I know I have cancer in my blood. It’s a life-threatening disease and it’s a serious illness” (Avi, 16).

The majority of the participants (13/19) stated they do not need or do not want more information about their illness than they already know: “I’m not interested in knowing more”; “I wouldn’t want to know more”; “It’s not urgent for me. I get along as it is”; “I don’t have to [know]. For me, it’s enough.”

The participants also talked about the negative effect of the information burden, which might be scary for children: “Too much information can cause fear” (Dan, 12). “They give the kids the least intimidating information. The scary information is not voiced. Some children want to know, and some don’t want to know” (Johnny, 12).

**FIGURE 1:**
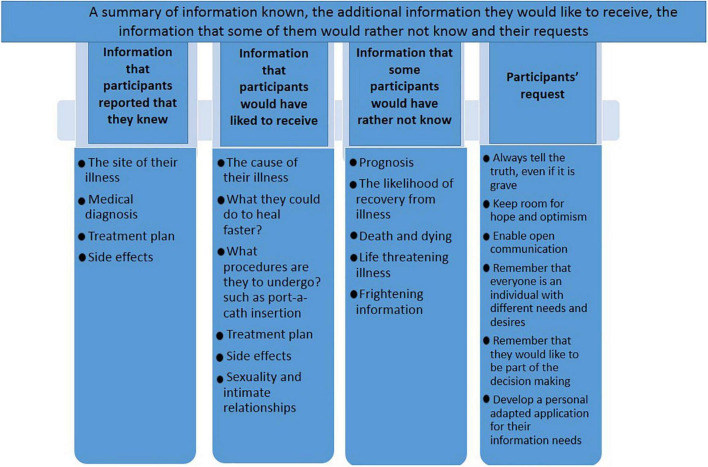
A summary of information known, the additional information they would like to receive, the information that some of them would rather not known and their request.

Some participants spoke about the frightening sides of knowing and still said that they prefer to know because for them it was reassuring: “[On the other hand] it might be better not to know. Because sometimes when you know you’re scared. And it can happen to you, just because of the thought about it. common side effects, but…. in spite of it, I prefer to know. I’d like to know more about what’s going on” (Jonathan, 16). No differences in age-related information needs were found in the current study. A summary of the information known, the additional information the participants would like to receive, the information some of them would rather not know, and the requests of the participants can be seen in Figure 1.

### The Opinions of Children and Adolescents About Truth-Telling

The participants spoke candidly about the merit of truth-telling to children. Esti (8.5), the youngest participant, wondered: “Why does the hair fall out? Why does the illness appear? And about the treatments and the pills. You need to say how long it will last; to tell children the truth from the beginning.” Samuel (18), the oldest participant, added: “I feel like I am told everything, and it is important for me to know every step of the treatment, including future treatment options.”

A few participants spoke about their feeling that information is being hidden from them: “At first, they didn’t tell me anything. I was told that this is a kind of flu. Then I was hospitalized. I saw different children. And I didn’t understand what this had to do with the flu. I inquired about it in more depth – What flu? What type of the flu? I asked the other kids what they were sick with. They told me it was blood cancer. If I could turn time back I’d like you to tell me the truth.” (Eli, 18) “I think I know the large part of it. If there are other things? I believe they don’t hide [things] from me” (Samuel, 18). Ron (16) said that he was satisfied with the information shared with him and stressed the importance of sharing: “Otherwise he/she might suspect that something is wrong.” However, he said that the degree of sharing depends on the age of the child. Participants shared the reasons why they thought it was important to tell the truth. “I think a child should always be told the truth. A child who is not told can be shocked and angry that they are not included” (Jonathan, 16).

Ruth (13) added: “I don’t like it when information is concealed from me. I want to be in control.” She explained that she would rather not know about medications and leave it to her parents, but if anesthesia is involved, she needs to know what is being done, because when under anesthesia she is not in control. Avi (16) preferred “to know the truth, even if it is difficult to say; it is better [to hear it] directly than to seek the information from other sources. I searched the internet, Google. I don’t think it’s good to search on Google – most things are frustrating.” He explained that he is an introverted person and the internet gave him the feeling that he had someone to talk to, but “even if a child is an introvert and does not ask for information, they should tell him.” Most of the participants mentioned the importance of being included. One participant even mentioned the importance of the informed consent form for chemotherapy and the need to show it to adolescents even when under 18: “I only recently found out that there is such a form [informed consent]… I would have felt more comfortable if they had shown it to me much earlier. I think every adolescent should insist on reading it (Eli, 18).”

### The Sources Children and Adolescents of Information and Support

As seen in Table 3, the participants identified several significant sources of information and support. The participants admitted that when they were diagnosed they had searched for information online. However, they no longer do so: “The internet was in the beginning. Today I ask doctors and nurses too” (Amir, 17); “I seek information from the doctors and nurses. I don’t look for information on the internet because it’s unreliable. It’s better to ask people rather than a computer” (Avi, 16). Four adolescents claimed that it is important to have accurate information and that a customized app can fulfill this need: “I think we should build a structured and organized application that would provide us with all the details that are relevant for us” (Amir, 17). Avi (16) also thought of that idea: “We need to learn a new and complex language and the information needs to be tailored and relevant for everyone. Developing a special app can help us a lot. It is important that we have no question marks remaining, but we do not always know what to ask or feel comfortable asking.”

**TABLE 3:** The sources of information and support for children and adolescents.

Source of information	

Provider	Interviewees’ responses
Internet	Was used only at the time of diagnosis Deemed as an unreliable source of information
Application	Non-existent. Was proposed as future development that could serve, as a suitable source of relevant information for them that could help them cope
Physicians	14 out of 19 participants perceived physicians as a primary source of information
Nurses	3 out of 19 participants perceived the nurses as a primary source of information
Parents	2 out of 19 participants perceived the parents as a primary source of information

**Source of support**	

**Provider**	**Interviewees’ responses**

Family and friends	Most of the participants talked about the support that comes from family and friends and how much it strengthens them
Volunteers	The good morale in the hospital department and the volunteers who come and spend time with them help them cope

Regarding the question from whom they received the information, 14 participants said that their primary source of information is physicians. Three participants said that their primary source of information is nurses and only two participants stated that their primary source of information is their parents.

Besides information as a helpful and supportive resource, the participants were asked what else helped them cope. Most of the participants noted the support of family and friends: “[What helps me are] conversations with dad, which gives me a view of more difficult situations” (David, 15). Two participants mentioned the good atmosphere in the [hospital] department as a source of support: “Always happy. The volunteers. raise morale” (Ron, 16); “[It is] always fun here [in the department]. So that’s good for me.” Esti (8.5) also mentioned that volunteers, friends, and family are those who most help cope with the disease.

When asked what helps them the most, the participants talked about how they maintain optimism, for example, Esti, (8.5) talked about the encouragement she receives from her surroundings: “The encouragement I get – that I’m good and I’m a champion.” She also spoke about the thought that cancer would not last forever: “…the thought that everything will [eventually come to an] end. It strengthens me.” Looking at cancer as a temporary situation was also how Amir (17) perceived it: “A child should know, remember, that it is only a [limited] period of time and that it will pass,” and in the same spirit, Ron (16) described how practical knowledge has helped him remain optimistic: “Knowing at each stage what to do and why. Cancer is not the end of the world. You should stay optimistic.” Samuel (18) revealed that he is encouraged by the thought of other children who are dealing with similar things and by those who have recovered from the disease: “To see other kids who are struggling heroically.” Edna (16) spoke about keeping her head up even in uncertain situations. She spoke about the need to be patient: “There is not always an immediate answer.”

## Discussion

The current study adds knowledge received from adolescents with cancer regarding providing information about their illness. The analysis of the transcribed conversations with the research participants uncovered several important themes that recurred among most of them. In this chapter, we shall discuss these themes and compare them to previous findings in the literature.

### The Need for Information of Children and Adolescents

Concerning knowledge about the disease, the participants knew the name of their disease and its site in their body and in this context, most did not feel that they need or would like to receive additional information. They were very interested in practical knowledge on the treatments *per se*, were familiar with the treatment protocol, and said that they would like to receive more information. The participants wanted to be more involved in decisions concerning the treatments and wanted the staff to see them as an inseparable part of the decision-making process. One participant even noted that, in his opinion, adolescents (under 18) should be asked to sign an informed consent form. During the interviews, the interpersonal diversity and complexity regarding the extent of the need for information about the treatments were conspicuous. The participants noted that receiving information helps them deal better with the disease and treatment protocol, however, excessive information is difficult for them and arouses fears and concerns. To meet the needs of the affected adolescents, the provision of information should be adapted to the individuals, their developmental stage, and their illness stage. Some said that information would lead to good and positive feelings such as confidence and trust in the medical staff and themselves, while for others, the same information would result in fear. Some claimed, furthermore, that even if certain information arouses concerns at an initial stage of the illness, at the end of the process, they see it as an advantage. The study shows that the participants saw the role of the medical staff as one that facilitates openness on various topics while maintaining sensitivity to the needs and wishes of the ill adolescent. One of the topics that occupy adolescents but was not freely expressed is related to intimacy, their sexual image, and sexual behavior. The two participants who initiated the conversation surrounding sexuality requested that the medical staff enable an open discourse on all possible topics, those that are easier to discuss but also those that are harder to talk about, such as sexuality. The difficulty to initiate conversations about sexuality is also supported by the study conducted by Veneroni et al. (2020).

### What Information Is of Less Interest?

An interesting issue that the adolescents did not want to talk about was related to the prognosis. The participants said explicitly that such information might weigh heavily on them and prevent them from feeling hope. Perhaps not discussing their prognosis allows them to cope by disregarding and denying it so that they can hold on to hope. This clear message arose repeatedly in the interview with most of the participants. A study conducted by Mack et al. (2018) among adolescents aged 15–29 showed different results regarding the importance of conversations about prognosis issues from the perspective of the patients. Their study found that the patients who reported having received more extensive prognostic information experienced greater trust in the oncologist, greater peace of mind, and less distress. Despite the positive perception associated with the discourse on prognosis, their study found differences between the chances of recovery reported by the physician and the patient, where patients were inclined to perceive the chance of recovery in a more positive light than the physicians. It may be concluded that patients find it difficult to talk about a gloomy prediction regarding their future. This assumption can explain the current finding showing that adolescents did not want to talk about their prognosis. Adolescents may be so anxious about the negative outcomes of their illness may be reluctant to deal with threatening issues such as the risk of dying, that they prefer not to talk about them at all. Creative ways of enabling a protected discourse on these sensitive topics should be considered. The discussion of sensitive topics should begin with listening. The medical team should ask adolescents about their experience and listen to their answers before bringing up any feeling or explanation. In addition, they should offer honest answers but always leave room for hope and consider their needs, preferences, and condition.

### Telling the Truth

During the interviews, the significance of honesty between the parents, medical staff, and the ill adolescent arose. Adolescents with cancer want information and want to know the truth. They sense when information is being concealed from them or when they are not included, and they become frustrated. The interviewees claimed that it is important to talk about anything the adolescent desires, and it is particularly important, to tell the truth, and not to conceal it, and certainly, not to lie. The participants suggested talking honestly about the situation even if it is grave. They claimed that the truth contributes to a sense of control even if it is hard to hear while concealing and not telling the truth lead to feelings of fear, loneliness, and distrust. It is evident from the findings that although everyone stated that the truth should be told, the age of the child has an important effect on how the truth is told, as it determines the determination of the child to deal with tough information. A 9-year old talked about the significance of telling the truth but claimed that words should be chosen carefully, while an 18-year-old emphasized that it is important for him to know everything – every stage of the treatment, and that he wants to be included in decisions that involve choosing between existing options. The need for age-adapted information is also supported by the literature review published by Raz et al. (2016) and Bibby et al. (2017). For example, it is important to choose the right words according to the age of the patient. Young children may better understand the meaning of the word “cancer” through images like “sick cells” or “soldiers in the body” as described by Esti (8.5) in the current study, while older children and adolescents may prefer to hear the words as they are such as “cancer,” “malignant,” etc.

### Information Sources

Regarding information sources, the study showed that all the participants claimed that they would like to receive information from people – parents, doctors, and nurses. This information is important for them to feel involved and included. Similar to Coyne et al. (2016), in the current study, the participants stated that they trust their parents to provide them with the selected information that they should know. The parents constitute their anchor and support system and they appreciate them as the mediators of information who adapt it for their children so that they will be able to deal with it. A meta-analysis conducted by Yamaji et al. (2020) revealed similar findings, which highlighted that children perceived their parents as reliable suppliers of information and as an important support resource.

Beyond the parents as information suppliers and to confirm the information they possess, adolescents also ask the staff. This finding is consistent with other studies showing that health care providers are perceived as the primary source of information for AYA (Kent et al., 2016). Despite the wish of the adolescents to receive information from nurses, the interviews show that although the nurses are in close and continuous contact with the adolescents and their families, they are less perceived by adolescents as the main information resource.

Only a few of the interviewees used the internet as a source of information, and they did so, particularly, in the initial stage. The interviewees said that in the course of their illness, the further they got from their diagnosis, they avoided searching for information on the internet both because they received the information they need from significant others (parents and staff) and because the information on the internet is general and is not tailored to their specific state or condition which might lead to feelings of fear, insecurity, and uncertainty. Even participants who would search for information about their disease on the internet immediately after diagnosis quickly realized that this information was too general, did not suit them personally, and was sometimes threatening and harsh. Research conducted among cancer survivors aged 18–39 years revealed similar findings regarding the vast and extensive information available on the internet. The participants who searched for healthy lifestyle information on the internet said that there was too much information on the internet and that the information they found was not tailored to their unique challenges and needs as AYA cancer survivors. One AYA cancer survivor stated, “Everybody is different, so there would need to be some specialized programs based on your diagnosis and what’s recommended” (Mooney et al., 2017).

The current study shows the need for advanced technology in the service of information that is personally adapted to the patients. A similar finding arose in a Canadian study held among 33 adolescents, aged 12–18 years, with chronic illnesses. The participants claimed, similar to the findings of the current study, that they are interested in developing a personally adapted application that would help them cope and give them access to trustworthy, professional, and authorized information (Kohut et al., 2017).

### Clinical Implications

This study includes a sample of 19 eligible Israeli adolescents who completed a psychological interview consisting of 15 open-ended questions used in previous studies. The main results showed that whereas the participants knew enough about the course of their illness, they were not informed about its etiology. They were very interested in practical knowledge about the treatments *per se*, were familiar with the treatment protocol, and would like to receive more information about the treatments and sexuality and intimacy. The participants reported a lack of knowledge regarding these issues and felt a sense of discomfort talking about them. The adolescents would like the medical professionals to leave them with no question marks and to provide them with the information they need, especially because they do not want to use the internet as a source of information due to the profusion of stressful and inadequate information it contains. The findings also revealed that adolescents want to receive information and to be involved in decision making and yet want the information they receive to be positive and to leave them with optimism and hope for the future.

The study indicates that similar to that known from the literature, adolescents are indeed a unique population that needs special attention and special understanding due to the many physical, mental, and social changes typical in adolescence, together with the traumatic crisis surrounding a life-endangering, scary, and lengthy illness. The knowledge, understanding, and open and adapted channels of communication concerning the disease and its treatment between the adolescent, the family, and the medical staff, are crucial for the ability of the adolescent and their family to handle the hardships caused by the treatment, the illness, and the recovery process. The research findings help the staff understand the communication and information needs of adolescents who are at a complex stage of their development, between childhood and maturity. They are no longer children but have not yet matured as adults. At this stage of their development, they seem to require a balanced combination of the parental protection they need as children and the desire to know, share, and control what they need as adults. The adolescents seek to be involved but also seek a type of protection during conversations related to their medical condition. They need to be part of a positive discourse that will leave room for hope. It is important for them to hear, as shown by Jalmsell et al. (2016), that the illness is a passing phase in their life, and that the future awaits them.

The following recommendations were derived from the contents raised by the interviewees in the study. These recommendations might advance treatment, as well as provide access to information for adolescents with cancer:

1. The medical staff must include the patient and actively provide access to information, while demonstrating honesty and sensitivity.
2. Initiated conversations can take place even when not actively requested by the adolescents, to provide them with information that is as inclusive and accurate as possible.
3. The issue of age and interpersonal differences that lead to different desires for information by adolescents should be taken into account before providing information. It is desirable to examine and explore whether there are topics that they are afraid or ashamed to talk about.
4. Before initiating a conversation, it would be desirable to ask: “What do you already know and what would you like to know more about?”
5. Presenting an informed consent form for chemotherapy to patients should be considered even in the case of adolescents who have not yet reached the legal age of informed consent. Full inclusion might contribute to a sense of belonging and involvement.
6. Finding the resources to develop a personally adapted application that adolescents can use to detect where they are on the health and illness sequence and to receive accurate information when desired, should be considered. This can involve developing a type of “Onco-Waze” (road map). This application might reduce concerns and uncertainty and increase knowledge and a sense of security and partnership.
7. Parents should be instructed, from the stage of diagnosis, to share adapted information and to avoid saying anything that does not arouse trust.
8. It must be clarified to parents that adolescents are capable of dealing with tough information positively and that they prefer this over dealing with a lack of information or incorrect information.
9. A supportive environment contributes to positive feelings among adolescents. Try to produce and encourage social support through friends, family, and volunteers.
10. Staff should be trained to care for the population of adolescents with cancer by providing knowledge about this complex developmental stage, in times of health and sickness. By training the staff, it is possible to provide a more adjusted response for this population and thus, help them cope during the treatment period and subsequently when resuming their routine.
11. Similar studies should be conducted to facilitate evidence-based research on how information is provided and the type of information that is suitable for adolescents with cancer.

### Research Limitations

The research findings should be interpreted taking into account several limitations. One is related to the small sample size, which may be a limitation on the conclusion. However, it should be noted that childhood cancer is a rare disease and of all children with cancer, adolescents constitute an even smaller percentage. In addition, the age and gender of the research participants were heterogeneous, and in each age group, there was a limited number of interviewees. This may have inhibited finding significant differences for each age and gender group with regards to the information needs or different perceptions of adolescents regarding the issues that arose in the study. Another limitation is related to the religiosity of the interviewees. Most of the research participants defined themselves as religious, and this can affect the information-related issues that occupy them. The religiosity of the participants might explain why only two interviewees (who were secular) raised the need for information on sexuality. The nationality of the interviewees may constitute a further limitation of the study. All the interviewees belonged to a single nationality. Further research that will also include sick Muslim and Christian participants on different levels of religiosity may be able to examine cultural differences in the information needs of adolescents with cancer in Israel. Another limitation is related to the fact that the participants were treated at a single hospital in Central Israel. Examining the information needs of adolescents treated at other hospitals would provide a more comprehensive picture.

## Conclusion

In conclusion, adolescents with cancer need trustworthy information and prefer to receive it from a human source rather than from the internet, such as Google, etc. Not being told the truth can arouse negative feelings of fear and loneliness in adolescents. Thus, medical professionals should operate in sensitive ways to provide adolescents with access to information on various subjects, including sexuality. Open communication and trust relations between the medical staff, parents, and adolescents may be a key to enhancing the resilience and well-being of adolescents (Mack et al., 2018; Yamaji et al., 2020). Besides human sources of information, there is room to consider developing technologies that will provide adolescents with personalized information appropriate for their needs, which will be available to them in any situation and at any time.

## Data Availability Statement

The original contributions presented in the study are included in the article/supplementary material, further inquiries can be directed to the corresponding authors.

## Ethics Statement

The studies involving human participants were reviewed and approved by the Helsinki Committee at the Rabin Medical Center. Written informed consent to participate in the study was provided by the participants’ legal guardian/next of kin.

## Author Contributions

All authors listed have made a substantial, direct and intellectual contribution to the work, and approved it for publication.

## Conflict of Interest

The authors declare that the research was conducted in the absence of any commercial or financial relationships that could be construed as a potential conflict of interest.

## Publisher’s Note

All claims expressed in this article are solely those of the authors and do not necessarily represent those of their affiliated organizations, or those of the publisher, the editors and the reviewers. Any product that may be evaluated in this article, or claim that may be made by its manufacturer, is not guaranteed or endorsed by the publisher.
